# Outcome of in- and out-of-hospital cardiac arrest survivors with liver cirrhosis

**DOI:** 10.1186/s13613-017-0322-1

**Published:** 2017-10-06

**Authors:** Kevin Roedl, Christian Wallmüller, Andreas Drolz, Thomas Horvatits, Karoline Rutter, Alexander Spiel, Julia Ortbauer, Peter Stratil, Pia Hubner, Christoph Weiser, Jasmin Katrin Motaabbed, Dominik Jarczak, Harald Herkner, Fritz Sterz, Valentin Fuhrmann

**Affiliations:** 1 Department of Intensive Care Medicine, University Medical Center Hamburg-Eppendorf, Martinistraße 52, 20246 Hamburg, Germany; 2 Department of Emergency Medicine, Medical University of Vienna, Vienna, Austria; 3 Division of Gastroenterology and Hepatology, Department of Internal Medicine 3, Medical University of Vienna, Vienna, Austria

**Keywords:** Cardiac arrest, Cirrhosis, Acute-on-chronic liver failure, Multiple organ failure, Intensive care unit

## Abstract

**Background:**

Organ failure increases mortality in patients with liver cirrhosis. Data about resuscitated cardiac arrest patients with liver cirrhosis are missing. This study aims to assess aetiology, survival and functional outcome in patients after successful cardiopulmonary resuscitation (CPR) with and without liver cirrhosis.

**Methods:**

Analysis of prospectively collected cardiac arrest registry data of consecutively hospital-admitted patients following successful CPR was performed. Patient’s characteristics, admission diagnosis, severity of disease, course of disease, short- and long-term mortality as well as functional outcome were assessed and compared between patients with and without cirrhosis.

**Results:**

Out of 1068 patients with successful CPR, 47 (4%) had liver cirrhosis. Acute-on-chronic liver failure (ACLF) was present in 33 (70%) of these patients on admission, and four patients developed ACLF during follow-up. Mortality at 1 year was more than threefold increased in patients with liver cirrhosis (OR 3.25; 95% CI 1.33–7.96). Liver cirrhosis was associated with impaired neurological outcome (OR for a favourable cerebral performance category: 0.13; 95% CI 0.04–0.36). None of the patients with Child–Turcotte–Pugh (CTP) C cirrhosis survived 28 days with good neurological outcome. Overall nine (19%) patients with cirrhosis survived 28 days with good neurological outcome. All patients with ACLF grade 3 died within 28 days.

**Conclusion:**

Cardiac arrest survivors with cirrhosis have worse outcome than those without. Although one quarter of patients with liver cirrhosis survived longer than 28 days after successful CPR, patients with CTP C as well as advanced ACLF did not survive 28 days with good neurological outcome.

**Electronic supplementary material:**

The online version of this article (doi:10.1186/s13613-017-0322-1) contains supplementary material, which is available to authorized users.

## Background

Patients with liver cirrhosis [1] and organ failure admitted to intensive care units (ICU) have high morbidity and mortality [2, 3]. Mortality rates of up to 80% are reported in critically ill cirrhotic patients, progressively increasing with the number of organs failing [4–6]. Recently, chronic liver failure-SOFA (CLIF-SOFA) score [3] was developed as a tool for risk stratification in patients with cirrhosis and acute-on-chronic liver failure (ACLF) [2, 3].

Cardiac arrest (CA) can be the consequence of or lead to multiple organ failure. It is one of the leading causes of death in many parts of the world. Every year estimated 375,000–700,000 citizens are suffering CA in Europe and the USA [7, 8] and receive cardiopulmonary resuscitation (CPR). Patients who achieve return of spontaneous circulation (ROSC) following CA have high morbidity and mortality mainly due to cerebral and cardiac dysfunction that accompany whole-body ischaemia and reperfusion [9]. These disabilities can lead to the post-CA syndrome, which is defined as multiple organ failure after CA. Despite advances in critical and emergency care, survival rates after in-hospital cardiac arrest (IHCA) and out-of-hospital cardiac arrest (OHCA) are generally poor and varying greatly for OHCA between 8–16% [10, 11] and for IHCA 14–23% [12–14].

Data on occurrence and outcome of CA in patients with liver cirrhosis are not available. Therefore, the aim of the study was to investigate cause and outcome in patients with liver cirrhosis after CA and ROSC compared to a large cohort of patients with CA and ROSC without liver cirrhosis.

## Methods

This study was based on a prospectively maintained registry at the Emergency Department of the Medical University of Vienna. This registry was approved by the ethics committee of the Medical University of Vienna. Due to the observational character of the study, informed consent was waived. The study was performed between January 2005 and January 2012. All consecutive patients admitted to the Emergency Department of the Medical University Vienna after CA and ROSC were included in the analysis. CPR and post-CA care were performed in accordance with the European Resuscitation Council guidelines [15, 16]. The data were collected prospectively according to Utstein-style guidelines [17, 18]. Patients suffering from OHCA were treated by the Viennese two-tier EMS system, featured by an EMS physician and paramedics; the EMS system was described previously in detail [19, 20]. No-flow time was defined as the time period from onset of CA to the start of resuscitation efforts. Low-flow time was defined as the time period from the start of resuscitation efforts until ROSC. Time to ROSC was defined as time from onset of CA until ROSC. CA survivors were followed prospectively for at least 1 year after admission to the emergency department for assessment of survival and neurological outcome. Rates of 28-day mortality, 6-month mortality and 1-year mortality were assessed on site or by contacting the patients or their attending physicians. Cerebral function and overall performance were assessed on admission and after 28 days, 6 months and 1 year, by clinical visits, by physicians on site or contacting the attending physician, the patients or the family of the patient directly by telephone. Cerebral performance categories (CPC) [21] and overall performance categories (OPC) scales were used to assess neurological and overall outcome. A CPC/OPC score of 1–2 was defined as favourable neurological/overall outcome, such as 3–5 as unfavourable. The primary outcome was good neurological survival (CPC 1/2) after 6 months; our secondary outcome was overall mortality after 1 year.

Routine laboratory assessment including coagulation and liver function parameters was performed on daily basis. Furthermore, aetiology of CA (cardiac and non-cardiac origin like pulmonary, traumatic, cerebral, septic, intoxication, drowning, hypothermia, unclear and others) and underlying diseases were assessed and documented.

Severity of illness was evaluated in all patients using Sequential Organ Failure Assessment (SOFA) score [22] and Simplified Acute Physiology Score (SAPS II) [23]. Charlson comorbidity index (CCI) [24] was calculated in all patients. For patients with liver cirrhosis, model of end-stage liver disease score (MELD) [25], Child–Turcotte–Pugh (CTP) score [26] and CLIF-SOFA score [3] were calculated on admission, and CLIF-SOFA was additionally calculated 24 and 48 h after ROSC.

All patients were screened for signs of liver cirrhosis. Presence of liver cirrhosis was defined via histology, if available, otherwise by a combination of clinical characteristics (ascites, spider angiomata, caput medusa), laboratory and radiological findings (typical morphological changes of the liver, sings of portal hypertension, etc. in ultrasonography or computed tomography scanning).

### Statistical analysis

Data are presented as count and relative frequency or median and 25–75% interquartile range (IQR). We tabulated clinical variables according to liver cirrhosis status and used Chi-squared, Fisher exact or Mann–Whitney *U* test for hypothesis testing as appropriate. The prognostic factor of interest was liver cirrhosis, and we used logistic regression to estimate the effect on neurological intact survival. The dependent variable was favourable neurological survival (best CPC 1 or 2; yes vs. no). In a multivariable logistic regression model, we entered liver cirrhosis as main covariable and age, sex, OHCA, witnessed CA, time to ROSC, presence of shockable rhythm, cardiac cause of CA, mechanical ventilation, SOFA on admission, initiation of MTH, CCI and cumulative adrenaline dose as other covariates to the model. To allow for potentially non-random missing data for time to ROSC caused by unwitnessed cardiac arrest, we categorised this variable for 0–4, 5–12, 13–24, 25–44, 45 + minutes as well as ‘missing’ as the sixth category. We used a similar model to estimate the associations with mortality at one year as the outcome. In all models, we tested for linear effects, first-order interactions and model fit using the likelihood ratio test. Survival function estimates were calculated using Kaplan–Meier method and were compared by the log-rank test. Statistical analysis was conducted using Stata 14 (StataCorp, College Station, TX) and IBM SPSS Statistics version 23.0 (IBM Corp., Armonk, NY). Generally, a *p* value < 0.05 was considered statistically significant.

## Results

### Study population

In total, 1068 patients (72% male, median age 61 years) after CA and ROSC were included in this study. Forty-seven (4%) of these patients had liver cirrhosis. Main cause of CA was cardiac in 678 (63%) patients of the total cohort. A total of 798 (75%) patients suffered CA out of hospital. Patients with liver cirrhosis had a significantly higher underlying non-cardiac cause compared to patients without cirrhosis. Median SOFA, SAPS II and CCI on admission were significantly higher in patients with liver cirrhosis. Sex, age, height and weight were distributed equally between both groups. Detailed characteristics of the study population are given in Table 1. Liver function and coagulation parameters were significantly different between patients with and without cirrhosis on hospital admission as illustrated in Additional file 1: Table S1.

**Table 1:** Patients’ characteristics of the study population at admission stratified according to the presence of cirrhosis

Parameters	All patients (*n* = 1068)	Cirrhosis (*n* = 47)	No cirrhosis (*n* = 1021)	*p* value
Age, years *median; IQR*	61 (50–72)	62 (51–67)	61 (50–72)	0.92
Male, *n %*	765 (72)	35 (74)	730 (72)	0.66
Weight, kg *median; IQR*	80 (70–90)	80 (69.5–93)	80 (70–90)	0.72
Height, cm *median; IQR*	175 (168–180)	175 (165–180)	175 (168–180)	0.59
SOFA admission, pts *median; IQR*	9 (6–12)	11 (7.5–13)	9 (6–12)	< 0.05
SAPS II admission, pts *median; IQR*	80 (74–88)	87 (77.5–100)	80 (73–87)	< 0.001
Charlson comorbidity index, pts. *median; IQR*	1 (0–3)	4 (2.5–6)	1 (0–2)	< 0.001
Cause of arrest, *n %*				
Cardiac	678 (63)	21 (45)	657 (64)	< 0.05
Out of hospital, *n %*	798 (75)	31 (66)	767 (75)	0.16
Invasive mechanical ventilation, *n %*	850 (80)	35 (74)	815 (80)	0.37
Before cardiac arrest—normal CPC, *n %*	1043 (98)	45 (96)	998 (98)	0.45
Before cardiac arrest—normal OPC, *n %*	973 (91)	39 (83)	934 (91)	0.09
Ischaemic time, min median; IQR*				
No flow	0 (0–3)	0 (0–3.5)	0 (0–3)	0.49
Low flow	13 (4–25)	11 (3–23)	13 (4–25)	0.51
Time to ROSC	16 (5–30)	15 (3–27)	16 (5–30)	0.42
Epinephrine cumulative (mg) *median; IQR*	3 (1–4)	3 (1–5.5)	3 (1–4)	< 0.001
Witnessed cardiac arrest, *n %*	921 (86)	42 (89)	879 (86)	0.56
Initial rhythm, *n %*				
VT/VF	550 (51)	10 (21)	540 (53)	< 0.001
PEA/asystole	465 (44)	35 (75)	430 (42)	
Other/unknown	53 (5)	2 (4)	51 (5)	0.82
Defibrillation, *n %*	646 (60)	14 (30)	632 (62)	< 0.001
Therapeutic hypothermia, *n %*	666 (62)	18 (38)	648 (63)	< 0.001

*SOFA* Sequential Organ Failure Assessment, *SAPS* Simplified Acute Physiology Score, *CPC* cerebral performance categories, *OPC* overall performance categories, *ROSC* return of spontaneous circulation, *VT* ventricular tachycardia, *VF* ventricular fibrillation, *PEA* pulseless electrical activity

* Overall (*n* = 926), cirrhosis (*n* = 41), no cirrhosis (*n* = 1021)

### Characteristics of patients with liver cirrhosis

Main underlying aetiology of liver cirrhosis was alcoholic liver disease (*n* = 35, 74%) followed by viral hepatitis (*n* = 6, 13%) and others (*n* = 6, 13%). Child–Turcotte–Pugh (CTP) class prior to admission was A in 17 (36%), B in 17 (36%) and C in 13 (28%) patients. Hepatocellular carcinoma (HCC) was present in three patients. Three patients had transjugular intrahepatic portosystemic shunt (TIPS), and one patient was listed for liver transplantation prior to occurrence of CA. No patient had liver transplantation during follow-up. Aetiology of CA was cardiac (*n* = 21, 45%), variceal bleeding (*n* = 6, 13%), sepsis (*n* = 5, 11%), respiratory insufficiency (*n* = 5, 11%), electrolyte disturbances (*n* = 4, 9%) and other causes (*n* = 6, 13%). Detailed data are illustrated in Table 2.

**Table 2:** Characteristics of patients with cirrhosis stratified according to good 28-day outcome and bad 28-day outcome

Parameters	Overall (*n* = 47)	Good 28-day outcome (*n* = 9)	Bad 28-day outcome (*n* = 38)	*p* value*
Aetiology of cirrhosis, *n %*				0.33
Alcoholic	35 (74)	7 (78)	28 (74)	
Viral	6 (13)	2 (22)	4 (10)	
Other (cryptogenic, cardiac, etc.)	6 (13)	0 (0)	6 (16)	
Hepatocellular carcinoma, *n %*	3 (6)	0 (0)	3 (8)	0.38
Liver TX during follow-up, *n %*	0 (0)	0 (0)	0 (0)	
TIPS, *n %*	3 (6)	0 (0)	3 (8)	0.38
CLIF-SOFA—admission *median; IQR*	10 (6–12.5)	4 (3–4)	11 (9–13)	< 0.001
CLIF-SOFA—24 h *median; IQR*	10 (4.5–14)	4 (2–7)	12 (8.25–15.5)	0.01
CLIF-SOFA—48 h *median; IQR*	7 (2–12.5)	1 (1–7)	11 (6–13.75)	0.01
CTP—before admission, *n %*				0.05
A	17 (36)	6 (67)	11 (29)	
B	17 (36)	3 (33)	14 (37)	
C	13 (28)	0 (0)	13 (34)	
CTP points—before admission *median; IQR*	7 (5.5–10)	6 (5–7)	8 (6–11)	0.03
MELD—admission *median; IQR*	19 (10.5–24)	10 (9–10)	21 (14–24)	< 0.001
SOFA—admission *median; IQR*	11 (7.5–13)	4 (3–7)	12 (10–13.75)	< 0.001
SAPS II—admission *median; IQR*	87 (77.5–100)	66 (66–75)	92 (85–102.75)	< 0.001
Ascites—before admission, *n %*				0.33
None	15 (32)	4 (44)	11 (29)	
Mild	25 (53)	5 (56)	20 (53)	
Severe	7 (15)	0 (0)	7 (18)	
HE—before admission, *n %*				0.22
None	25 (53)	7 (78)	18 (47)	
I–II	17 (36)	2 (22)	15 (40)	
III–IV	5 (11)	0 (0)	5 (13)	
ACLF—on admission, *n* *%*				< 0.001
Grade 1	11 (23)	1 (11)	10 (26)	
Grade 2	11 (23)	0 (0)	11 (30)	
Grade 3	11 (23)	0 (0)	11 (30)	
No ACLF	14 (30)	8 (89)	6 (16)	
ACLF—during follow-up, *n* *%*				< 0.001
Grade 1	1 (2)	1 (11)	0 (0)	
Grade 2	3 (6)	1 (11)	2 (5)	
Grade 3	0 (0)	0 (0)	0 (0)	
No ACLF	10 (21)	6 (67)	4 (11)	
Ischaemic time, min *median; IQR*				
No flow	0 (0–3.5)	0 (0–0)	0 (0–5)	< 0.01
Low flow	11 (3–23)	1 (1–3)	16 (8–23.5)	0.06
Time to ROSC	15 (3–27)	1 (1–3)	19.5 (11–28)	< 0.05
Initial rhythm, *n %*				
VT/VF	10 (21)	3 (33)	7 (18)	0.569
PEA/Asystole	35 (75)	4 (45)	31 (82)	< 0.05
Other/unknown	2 (4)	2 (22)	0 (0)	0.305
Witnessed cardiac arrest, *n %*	42 (89)	9 (100)	33 (87)	0.118
Defibrillation, *n %*	14 (30)	3 (33)	11 (29)	0.569
Therapeutic hypothermia, *n %*	18 (38)	1 (11)	17 (45)	< 0.01

*CPC* cerebral performance categories, *TX* transplantation, *TIPS* transjugular intrahepatic portosystemic shunt, *CLIF-SOFA* chronic liver failure-Sequential Organ Failure Assessment, *CTP* Child–Turcotte–Pugh, *MELD* model for end-stage liver disease, *SOFA* Sequential Organ Failure Assessment, *SAPS* Simplified Acute Physiology Score, *HE* hepatic encephalopathy, *ACLF* acute-on-chronic liver failure

* CPC 1/2 versus CPC 3/4 or mortality

CLIF-SOFA on admission, 24 and 48 h following hospital admission, and MELD, SOFA and SAPS II on admission and CTP score prior to CA were significantly higher in patients with unfavourable neurological outcome or mortality within 28 days.

Thirty-three patients (70%) had ACLF on admission. Four (29%) out of 14 patients without ACLF on admission developed ACLF within 48 h after ROSC. Two (4%) of the patients had ACLF prior to admission. ACLF grades 1, 2 and 3 were present in 12 (32%), 14 (38%) and 11 (30%) patients, respectively.

### CPR-specific data

The majority of patients of the total cohort (75%) suffered OHCA. This rate did not differ significantly in patients with and without cirrhosis. Cardiac arrest was witnessed in 921 (86%) patients. No-flow time was median 0 (IQR 0–3) minutes, low-flow 13 (IQR 4–25) minutes and time to ROSC 16 (IQR 5–30) minutes, which did not differ significantly between patients with and without cirrhosis. Initial shockable rhythm (VT/VF) was significantly more frequent present in patients without cirrhosis. Accordingly, frequency of defibrillation during CPR was significantly lower in patients with liver cirrhosis. Furthermore, cumulative epinephrine dosage during CPR was significantly higher in patients with cirrhosis. First measured lactate levels were significantly higher in patients with liver cirrhosis. Table 1 and Additional file 1: Table S3 illustrate the detailed CPR data.

### Functional outcome and survival after CA

**Fig. 1:**
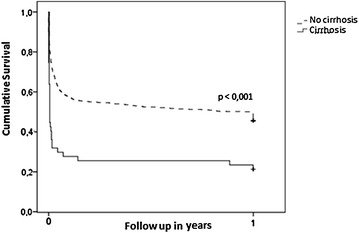
Survival after ICU admission for cardiac arrest according to the presence and absence of cirrhosis estimated by Kaplan–Meier method

Almost all patients both with and without cirrhosis showed a normal CPC and OPC score prior to occurrence of CA as given in Table 1. Rate of favourable neurological outcome was 19% after 28 days, 6 months and 1 year in patients with cirrhosis, compared to 47% after 28 days and 6 months and 43% after 1 year in patients without cirrhosis, respectively. Rate of favourable neurological outcome in CA survivors after 6 months was significantly lower in patients with liver cirrhosis (19 vs. 47%, crude OR 0.26; 95% CI 0.13–0.53). This association continued to be statistically significant after adjustment for covariables (multivariable-adjusted OR 0.13; 95% CI 0.04–0.36, see Table 3). Mortality in patients with cirrhosis was significantly higher compared to patients without cirrhosis (74, 77 and 79% versus 41, 48 and 50% after 28 days, 6 months and 1 year, respectively; OR for 1-year mortality 3.69; 95% CI 1.82–7.51). Figure 1 demonstrates the survival of patients with and without cirrhosis. Cirrhosis was an independent risk factor for 1-year mortality in multivariate regression analysis (multivariable-adjusted OR 3.25; 95% CI 1.33–7.96, see Table 4).

**Table 3:** Logistic regression model for factors associated with good neurological outcome (CPC 1/2 vs. > 2 or deceased)

Parameter	OR (95% CI)	*p* value
Cirrhosis (yes vs. no)	0.13 (0.04–0.36)	< 0.001
Age (years)	0.96 (0.96–0.98)	< 0.001
Time to ROSC (per category)*	0.56 (0.47–0.67)	< 0.001
Shockable rhythm (yes vs. no)	2.16 (1.45–3.23)	< 0.001
Intubated on admission (yes vs. no)	0.17 (0.09–0.34)	< 0.001
SOFA admission (per category)*	0.73 (0.64–0.82)	< 0.001
Cardiac cause (yes vs. no)	1.73 (1.17–2.58)	0.01
Epinephrine cumulative dose (per mg)	0.91 (0.85–0.98)	0.01
Witnessed cardiac arrest (yes vs. no)	0.62 (0.32–1.23)	0.17
Mild therapeutic hypothermia (yes vs. no)	1.23 (0.80–1.90)	0.36
Out-of-hospital cardiac arrest (yes vs. no)	0.89 (0.58–1.38)	0.61
Male (vs. female)	0.92 (0.65–1.31)	0.65
Charlson comorbidity index (per category)*	0.97 (0.84–1.12)	0.67

*OR* multivariable-adjusted odds ratio, *CI* confidence interval, *ROSC* return of spontaneous circulation, *SOFA* Sequential Organ Failure Assessment, *mg* milligram

* Time to ROSC categories: 0–4, 5–12, 13–24, 25–44, 45 + min, or missing; SOFA categories (score): 5, 6–8, 9–10, 11–12, 12 +, or missing; Carlson comorbidity categories: 0, 1, 2 + 3, 4 +, or missing

**Table 4:** Logistic regression model for factors associated with 1-year mortality

1-year mortality	OR	(95% CI)	*p* value
Age	1.05	(1.03–1.06)	< 0.001
Time to ROSC*	1.57	(1.32–1.87)	< 0.001
Shockable rhythm	0.45	(0.31–0.67)	< 0.001
Tube admission	2.33	(1.35–4.03)	< 0.001
SOFA admission*	1.36	(1.20–1.54)	< 0.001
Cirrhosis	3.25	(1.33–7.96)	0.01
Charlson comorbidity index*	1.19	(1.03–1.37)	0.02
Epinephrine cumulative	1.07	(1.01–1.15)	0.03
Male	1.41	(1.00–1.98)	0.05
Cardiac cause of CA	0.68	(0.46–1.00)	0.05
Mild therapeutic hypothermia	0.68	(0.45–1.05)	0.09
OHCA	0.99	(0.66–1.50)	0.98
Witnessed CA	1.00	(0.51–1.94)	0.99

*OR* multivariable-adjusted odds ratio, *CI* confidence interval, *OHCA* out-of-hospital cardiac arrest, *CA* cardiac arrest, *ROSC* return of spontaneous circulation, *SOFA* Sequential Organ Failure Assessment

* Time to ROSC categories: 0–4, 5–12, 13–24, 25–44, 45 + min, or missing; SOFA categories (score): 5, 6–8, 9–10, 11–12, 12 +, or missing; Charlson comorbidity categories: 0, 1, 2 + 3, 4 +, or missing

**Fig. 2:**
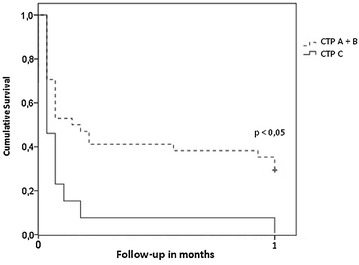
Probability of having a good neurological outcome after a cardiac arrest among cirrhotic patients according to Child–Turcotte–Pugh score estimated by Kaplan–Meier method

No patient with liver cirrhosis CTP C as well as no patient with HCC or pre-existing TIPS survived longer than 28 days following ROSC with good neurological outcome. Figure 2 demonstrates the survival of patients with favourable neurological outcome in cirrhosis CTP A + B versus CTP C.

Forty per cent (*n* = 4) of patients with cirrhosis without ACLF died within 28 days following ROSC or had unfavourable neurological outcome compared to 92% (*n* = 34) of patients with ACLF (*p* < 0.001). In detail, 83% (*n* = 10) of patients with ACLF grade 1, 93% (*n* = 13) with ACLF grade 2 and 100% (*n* = 11) with ACLF grade 3 had unfavourable neurological outcome or died within 28 days. Multiple organ failure as cause of death was observed in 30 patients with cirrhosis, one patient had cerebral herniation following hypoxic brain injury, and four patients died with palliative care following irreversible hypoxic brain damage.

Mild therapeutic hypothermia (MTH) was applied in 666 (62%) patients of the total cohort [18 (38%) patients with cirrhosis and 648 (63%) without cirrhosis (*p* < 0.001)]. Furthermore, MTH was significantly less frequent applied in patients with liver cirrhosis. In the cirrhosis population, patients with MTH were older, had lower SOFA, SAPS II, CLIF-SOFA and MELD score on admission and had significantly longer time to ROSC compared to patients without MTH. We could not observe bleeding or any other complication related to MTH. Twenty-eight-day mortality did not differ significantly between patients with cirrhosis and MTH versus no MTH (78 vs. 72%). Additional file 1: Table S2 illustrates the detailed data on MTH in patients with cirrhosis.

## Discussion

In our study, we analysed 1068 critically ill patients following CA and ROSC. Forty-seven of these patients suffered from liver cirrhosis. Presence of cirrhosis was associated with low rates of favourable neurological outcome and increased mortality. Highest rates of unfavourable functional outcome were found in advanced stages of cirrhosis and ACLF.

Cardiac arrest was witnessed in 921 (86%) patients of our cohort, and this is comparable to a previous publication [27]. Seventy-five per cent of the total cohort had OHCA. The first recorded rhythm was shockable in 52% of these patients which is comparable to the reported prevalence of 20–60% in the literature [10, 11, 28]. In contrast, shockable rhythms (35%) were found less frequent in IHCA in accordance with the literature (21–39%) [13, 14, 29]. In the total cohort, 1-year mortality was 50% and favourable functional outcome was observed in 43% of patients following CA. This high rate of favourable outcome may be the consequence of several circumstances. First, we included only patients following ROSC after CA. Accordingly, the rate of good functional outcome was comparable to other studies including only patients with ROSC [27, 30]. Second, we observed in average a short no-flow period in our cohort, which may contribute to the high rate of favourable functional outcome. Third, the vast majority (86%) of CAs was witnessed and we observed a shockable rhythm in about half of the patients.

We identified 47 (4%) patients with cirrhosis in our cohort. This finding is comparable to publications of critical illness, where prevalence of cirrhosis was about 4–7% in the general intensive care setting [31, 32]. These patients had significantly higher SOFA, SAPS II and CCI on hospital admission. Time to ROSC was comparable between patients with and without cirrhosis. However, we observed several significant differences in CA in patients with and without cirrhosis. First, a cardiac aetiology was less frequent and patients with cirrhosis were more likely to have a non-shockable initial ECG rhythm. Second, cumulative epinephrine dosage was higher during CPR and third defibrillations were less frequently performed in patients with cirrhosis. These differences seem to be mainly a consequence of the fact that patients with cirrhosis frequently developed CA following complications of cirrhosis like variceal haemorrhage, severe infection or severe electrolyte disturbances.

Rates of unfavourable functional outcome and mortality were significantly higher in patients with cirrhosis despite no-flow times that were comparable to patients without cirrhosis. This can be explained by the higher comorbidity rate, higher rate of non-cardiac cause and higher rate of non-shockable rhythm in patients with cirrhosis. Furthermore, multivariate regression analysis identified presence of cirrhosis *per se* as an independent predictor of unfavourable outcome.

Overall 38 (81%) patients with cirrhosis had unfavourable neurological outcome or died within 28 days following CA. These patients had a significant higher severity of liver disease and organ failure represented by CTP class, CLIF-SOFA, MELD and ACLF grade as illustrated in Table 2. ACLF was present in 33 (70%) cirrhotic patients at ICU admission following ROSC, and four patients developed ACLF within 48 h after admission. These patients had dramatically worse functional outcome: out of 37 patients with ACLF, 34 (92%) had unfavourable neurology or died within 28 days. Moreover, we observed significant differences in CA characteristics. Patients with cirrhosis and favourable 28-day outcome had a significantly lower no-flow time and time to ROSC and a significantly lower rate of non-shockable rhythm compared to patients with unfavourable neurological outcome or mortality. Furthermore, CA was witnessed in all cases in patients with favourable 28-day outcome. Ischaemic times, especially no flow, seem to be crucial for development of organ failure and unfavourable outcome in patients with cirrhosis. The higher rate of unfavourable 28-day outcome in patients with cirrhosis following CA and ROSC compared to critically ill patients with liver cirrhosis [3, 4, 33] may be explained mainly by the higher severity of illness at baseline in our cohort [4].

The post-CA phase is frequently complicated by the post-CA syndrome, a unique pathophysiological process involving multiple organs [9]. For instance, post-CA brain injury frequently complicates the post-CA phase and accounts for high morbidity and mortality [9, 33]. Factors like hyperglycaemia, impaired cerebral autoregulation as well as pre-existing cerebral impairment in the sense of hepatic encephalopathy could lead to further cerebral injury in patients with cirrhosis. Additionally, post-CA myocardial dysfunction and systemic ischaemia and reperfusion response are frequent findings and account for high morbidity and mortality after CA [15]. The severity of the post-CA syndrome varies according to duration and cause of CA [15]. In our cohort of patients with cirrhosis, death was mainly related to multiple organ failure (86%). Post-anoxic encephalopathy as solitaire cause of death was observed in a minority of patients with cirrhosis, only. Patients with cirrhosis seem to be more prone to organ impairment following CA. This seems to be a consequence of a higher vulnerability for new onset of organ failure and higher severity of illness during CA as discussed previously.

Mild therapeutic hypothermia, i.e. targeted temperature 32–36 °C for 24 h [15], is frequently used despite recent controversial findings as standardised post-CA care [27, 30, 34]. MTH was performed in 666 (62%) patients of our total cohort and in 18 (38%) patients with cirrhosis. Main reason for withholding MTH in cirrhosis was severely abnormal coagulation. A recent study of patients with cirrhosis demonstrated that abnormal coagulation parameters, especially fibrinogen and platelet counts, predict new onset of major bleeding in patients with cirrhosis at the ICU [33]. Data on bleeding complications due to MTH in patients with liver diseases are scarce. Two small case series [35, 36], a randomised controlled trial [37] and a retrospective study [38] of MTH in patients with acute liver failure did not observe an association of MTH and increased rate of bleeding complications. We could not observe new onset of bleeding or any other complication related to MTH in our cohort of critically ill patients with cirrhosis. Twenty-eight-day mortality did not differ significantly in patients with cirrhosis and MTH compared to patients with cirrhosis without MTH. This may be a consequence of the rather small number of patients with cirrhosis, the higher rate of OHCA and a significantly longer time to ROSC in the MTH group. In addition, this study was not powered to analyse the effect of MTH on prognosis in cirrhotic patients. Additional file 1: Table S2 illustrates the detailed data.

Multiple organ failure is associated with high mortality in patients with liver cirrhosis. ACLF is a dynamic condition, which can improve or worsen in a short period of time [39]. Early and repeated risk stratification may help and guide clinical decision making in this extremely sick population [40, 41]. Although our study is able to identify the population that is at highest risk of worst outcome (patients with advanced stages of ACLF and patients with cirrhosis CTP C), we do not believe that current knowledge is already sufficient to provide (i.e. score-based) cut-offs in regard to the decision whether to continue or to stop treatment due to futility. Rather, we are convinced that further therapeutic decisions, especially for withdrawal of care, must take individual patient based factors (i.e. severity of acute and chronic illness, patient’s wishes, etc.) into account. Furthermore, remaining treatment options (e.g. liver transplantation), course of the disease and severity of acute illness should be taken into account for further decisions by the attending physician. Future studies are warranted for end-of-life decisions in critically ill patients with cirrhosis [41, 42].

Our study has some limitations. The number of patients with cirrhosis is rather small. However, this is the first study investigating CA in patients with cirrhosis. Furthermore, this study was conducted in a medical intensive care setting. Thus, our data may not be transferable to surgical ICUs. Residual confounding arising from unmeasured covariates cannot be entirely excluded.

In conclusion, CA survivors with cirrhosis have worse outcome than those without pre-existent chronic liver disease. Although one quarter of patients with liver cirrhosis survived longer than 28 days after successful CPR, patients with CTP C as well as advanced ACLF did not survive 28 days with good neurological outcome.

## Additional file

**Additional file 1: Table S1.** Routine laboratory on admission. **Table S2.** Characteristics of cirrhotic patients with and without mild therapeutic hypothermia. **Table S3.** CPR-specific data according to in- and out-hospital cardiac arrest.

## Abbreviations

CA: cardiac arrest
ROSC: return of spontaneous circulation
ICU: intensive care unit
OHCA: out-of-hospital cardiac arrest
IHCA: in-hospital cardiac arrest
MTH: mild therapeutic hypothermia
CPC: cerebral performance categories
OPC: overall performance categories
SOFA: Sequential Organ Failure Assessment
SAPS: Simplified Acute Physiology Score
CCI: Charlson comorbidity index
MELD: model of end-stage liver disease
CTP: Child–Turcotte–Pugh
TIPS: transjugular intrahepatic portosystemic shunt
HCC: hepatocellular carcinoma
PCI: percutaneous coronary intervention
