# Supplementary material for: Outcome of in- and out-of-hospital cardiac arrest survivors with liver cirrhosis
Source: Ann Intensive Care. 2017 Oct 6;7:103. doi: 10.1186/s13613-017-0322-1 (PMC5630568; doi:10.1186/s13613-017-0322-1)
Supplement: Supplementary file 1 — Additional file 1: Table S1. Routine laboratory on admission. Table S2. Characteristics of cirrhotic patients with and without mild therapeutic hypothermia. Table S3. CPR-specific data according to in- and out-hospital cardiac arrest. [file 13613_2017_322_MOESM1_ESM.doc]

Table S1: Routine laboratory on admission

| **Parameters** | **All patients (n = 1068)** | **Cirrhosis (n = 47)** | **No cirrhosis (n = 1021)** | **p-Value*** |
| --- | --- | --- | --- | --- |
| **Hemoglobin** g/dl | 13.5 (11.8 – 14.6) | 11 (9.25 – 13.38) | 13.5 (11.95 – 14.6) | < 0.001 |
| **Hematocrit** % | 40.3 (36.2 – 43.8) | 33.8 (28.25 – 40.68) | 40.5 (36.7 – 43.9) | < 0.001 |
| **MCV** fl | 91.6 (88 - 95.2) | 92.95 (87.33 – 101.88) | 91.6 (88.1 – 95) | 0.289 |
| **Platlets (10^9^**/l) | 211 (162 – 260) | 154. 5 (113.5 – 221.75) | 213 (165 – 260) | < 0.001 |
| **Leukocytes** G/l | 12.36 (9.16 – 16.37) | 9.97 (7.51 – 16.17) | 12.37(9.29 – 16.43) | 0.143 |
| **Prothrombin Index** % | 85 (66 – 103) | 58.5 (37 – 82) | 86 (68 – 103) | < 0.001 |
| **INR** | 1.08 (0.99 – 1.24) | 1.31 (1.11 – 1.77) | 1.07 (0.99 – 1.21) | < 0.05 |
| **aPTT** s | 42.7 (34.4 – 67.5) | 44.05 (37.2 – 68.5) | 42.6 (34.4 – 67.3) | 0.570 |
| **Fibrinogen** mg/dl | 335 (269 – 411) | 356 (240.25 – 454.25) | 334 (270 - 409) | 0.666 |
| **d-Dimer** g/ml | 6.82 (2.85 -17.26) | 11.06 (2.43 – 16.48) | 6.32 (2.88 – 17.43) | 0.601 |
| **Sodium** mmol/l | 138 (135 – 140) | 134 (130.25 – 138) | 138 (136 – 140) | < 0.001 |
| **Potassium** mmol/l | 3.95 (3.55 – 4.47) | 4.44 (3.76 – 5.42) | 3.94 (3.54 – 4.45) | < 0.05 |
| **Chloride** mmol/l | 103 (101 – 106) | 100 (94 – 103) | 103 (101 – 106) | < 0.001 |
| **Calcium** mmol/l | 2.2 (2.08 – 2.3) | 2.16 (2.05 – 2.28) | 2.2 (2.09 – 2.3) | 0.440 |
| **Phosphate** mmol/l | 1.98 (1.38 – 2.7) | 1.95 (1.57 – 2.75) | 1.98 (1.37 – 2.7) | 0.734 |
| **Magnesium** mmol/l | 0.91 (0.81 – 1.04) | 0.94 (0.80 – 1.03) | 0.91 (0.81 – 1.04) | 0.771 |
| **Creatinine** µmol/dl | 116 (97 – 141) | 124 (94 – 223) | 116 (97 – 141) | < 0.05 |
| **BUN** mmol/l | 3,14 (2,44 – 4,22) | 3,77 (2,36 – 7,55) | 3,12 (2,43 – 4,14) | 0.055 |
| **Bilirubin** µmol/l | 9 (6 - 14) | 22 (10 – 34) | 9 (6 – 13) | < 0.001 |
| **Albumin** g/l | 36.9 (32.3 – 39.9) | 32.65 (26.03 – 36.73) | 37 (32.7 – 39.9) | < 0.001 |
| **Amylase** U/l | 71 (49 – 109) | 61.5 (39.5 – 95) | 71 (49 – 110) | 0.055 |
| **Lipase** U/l | 43 (27 – 69) | 44.5 (28.25 – 65.5) | 42.5 (27 – 69.3) | 0.993 |
| **Cholinesterase** kU/l | 6.15 (4.8 – 7.4) | 3.2 (1.66 – 4.98) | 6.2 (4.96 – 7.45) | < 0.001 |
| **AP** U/l | 83 (64 – 106) | 101.5 (77 – 124) | 82.5 (64 – 104) | < 0.05 |
| **ASAT** U/l | 99 (55 – 191) | 67.5 (42.25 – 172) | 101 (56 – 192) | < 0.05 |
| **ALAT** U/l | 73 (38 – 147) | 38.5 (23 – 65.75) | 77 (39 – 150) | < 0.001 |
| **GGT** U/l | 63 (34 – 115) | 102.5 (58 – 249.5) | 61 (34 – 110) | < 0.001 |
| **LDH** U/l | 391 (285 – 572) | 314.5 (264 – 482.25) | 393 (288 – 579) | < 0.05 |
| **CRP** mg/dl | 0.5 (0.2 – 1.4) | 1.44 (0.51 – 7.45) | 0.48 (0.2 – 1.32) | < 0.001 |
| **CK** U/l | 180 (102 – 356) | 120 (76.75 – 251) | 183 (103 – 357.5) | < 0.05 |
| **Troponin** ng/ml | 0.06 (0.02 – 0.21) | 0.04 (0.01 – 0.10) | 0.06 (0.02 – 0.21) | 0.701 |
| **proBNP** pg/ml | 469.8 (129.25 – 1790.75) | 2522 (530 – 4928) | 457.7 (126.78 – 1685.5) | < 0.05 |

**Cirrhosis vs. no cirrhosis, data is presented as median (IQR)*

*Abbreviations:* MCV, Mean corpuscular volume; INR, International normalized ratio; aPTT, **activated** **partial** **thromboplastin** **time**; s, seconds; mmol/l, millimoles per liter; g/dl, grams per deciliter; mg/dl, **milligrams** **per** **deciliter**; kU/l,kilounits per litre; U/l, units per liter; AP, a**lkaline phosphatase**; ASAT, **aspartate aminotransferase**; ALAT, a**lanin aminotransferase**; GGT, **gamma-glutamyl transferase**; LDH, lactate dehydrogenase; CRP, C-reactive protein; CK, Creatine kinase; proBNP, pro brain natriuretic peptide;

Table S2: Characteristics of cirrhotic patients with and without mild therapeutic hypothermia

| **Parameters** | **Cirrhosis (n = 47)** | **Mild therapeutic hypothermia (n = 18)** | **No mild therapeutic hypothermia (n = 29)** | ***p v*alue** |
| --- | --- | --- | --- | --- |
| **Age, years** *median, IQR* | 62 (51 – 67) | 64 (53 – 67) | 61 (51 – 67) | 0.497 |
| **Male,** *n %* | 35 (74) | 13 (72) | 22 (76) | 0,521 |
| **CTP before admission,** *n%*  A B C | 17 (36) 17 (36) 13 (28) | 6 (44) 9 (50) 3 (16) | 11 (38) 8 (28) 10 (35) | 0,237 |
| **MELD – admission** *median; IQR* | 19 (10.5 – 24) | 15,5 (7.8 – 19.8) | 21 (12 – 25) | < 0.05 |
| **SOFA – admission** *median; IQR* | 11 (7.5 – 13) | 10,5 (8.3 – 11.8) | 12 (7 – 14) | 0,362 |
| **SAPS II – admission** *median; IQR* | 87 (77.5 – 100) | 85,5 (78.5–94.8) | 91 (77 – 103) | 0,569 |
| **CLIF-SOFA – admission** *median; IQR* | 10 (6 – 12.5) | 9,5 (6.3 – 11) | 11 (6 – 14) | 0.124 |
| **Etiology of cirrhosis, *n %*** Alcoholic Viral Other (Cryptogenic, Cardiac, ..) | 35 (74) 6 (13) 6 (13) | 14 (78) 1 (6) 3 (16) | 21 (73) 5 (17) 3 (10) | 0,454 |
| **Out of hospital,** *n %* | 31 (66) | 15 (83) | 16 (55) | < 0.05 |
| **Initial rhythm.** *n %* VT/VF PEA/Asystole Other/Unknown | 10 (21) 35 (75) 2 (4) | 5 (28) 13 (72) 0 (0) | 5 (17) 22 (76) 2 (7) | 0.308  0.521  0.376 |
| **Time to ROSC,** minutes, *median; IQR* | 15 (3 – 27) | 23 (15 – 30) | 9 (2 – 21.5) | < 0.01 |
| **Cause of arrest,** *n %* Cardiac | 21 (45) | 7 (39) | 14 (48) | 0.373 |
| **Platelets (10^9^**/l), *median; IQR* | 155 (114–223) | 181 (134 – 240) | 134 (108 – 216) | 0.203 |
| **PI (%),** *median; IQR* | 59 (37 – 82) | 62 (41 – 96) | 51 (37 - 71) | 0.228 |
| **aPTT (**seconds) *median; IQR* | 44 (37 – 69) | 38 (34 – 45) | 49 (39 – 75) | < 0.05 |
| **Fibrinogen (**mg/dl) *median; IQR* | 356 (240 – 454) | 342 (261 – 403) | 365 (233 – 500) | 0.628 |
| **28d - mortality,** *n%* | 35 (74) | 14 (78) | 21 (72) | 0.379 |
| **CPC 1/2 – 28d,** *n%* | 9 (19) | 1 (6) | 8 (28) | 0.064 |
| **CPC 3/4 – 28d,** *n%* | 3 (6) | 3 (17) | 0 (0) | 0.150 |
| **OPC 1/2 – 28d,** *n%* | 9 (19) | 1 (6) | 8 (28) | 0.057 |
| **OPC 3/4 – 28d,** *n%* | 3 (6) | 3 (17) | 0 (0) | 0.054 |

*Abbreviations:* CTP, Child-Turcotte-Pugh; MELD, Model for End-stage Liver Disease; SOFA, Sequential Organ Failure Assessment; SAPS, Simplified Acute Physiology Score; CLIF-SOFA, Chronic Liver Failure-Sequential Organ Failure Assessment; VT, Ventricular Tachycardia; VF, Ventricular Fibrillation ; PEA, Pulseless electrical activity; ROSC, Return of spontaneous circulation; CPC, Cerebral Performance Categories; OPC, Overall Performance Categories;

Table S3: CPR specific data according In- and Out-Hospital cardiac arrest

| **Parameters** | **All** | **Cirrhosis - Overall** | **No-Cirrhosis**  **Overall** | **In-Hospital cardiac arrest** | | **Out-of-Hospital cardiac arrest** | | ***p* value*** |
| --- | --- | --- | --- | --- | --- | --- | --- | --- |
|  | (n = 1068) | (n = 47) | (n =1021) | Cirrhosis  (n = 16) | No Cirrhosis  (n = 254) | Cirrhosis  (n = 31) | No Cirrhosis  (n = 767) |  |
| **Ischemic time. min** *median; IQR*  No-flow  Low-flow  Time to ROSC | 0 (0 - 3) 13 (4 – 25) 16 (5 – 30) | (*n = 41*)  0 (0 – 3.5)  11 (3 – 23)  15 (3 – 27) | *(n = 885*)  0 (0 – 3)  13 (4 - 25)  16 (5 – 30) | (*n = 14*)  0 (0 – 0.5)  9 (2 – 14)  9.25 (2 – 14) | (*n = 240*)  0 (0 – 0)  5 (2 – 14)  5 (2 – 15) | (*n = 27*)  0 (0 – 56)  17 (6 – 24)  20 (6 – 28) | (*n = 645*)  1 (0 – 5)  17 (7 – 28)  20 (10 – 33) | 0.49  0.51  0.42 |
| **Witnessed cardiac arrest.** *n %* | 921 (86) | 42 (89) | 879 (86) | 15 (94) | 240 (94) | 27 (87) | 639 (83) | 0.56 |
| **Initial rhythm.** *n %*  VT/VF  PEA/Asystole  Other/Unknown | 550 (51) 465 (44) 53 (5) | 10 (21)  35 (75)  2 (4) | 540 (53)  430 (42)  51 (5) | 3 (19)  13 (81)  0 (0) | 93 (37)  151 (59)  10 (4) | 7 (23)  22 (71)  2 (6) | 447 (58)  279 (37)  41 (5) | < 0.001  < 0.001  0.82 |
| **Epinephrine cumulative (mg)** *median; IQR* | 3 (1 – 4) | 3 (1 – 5.5) | 3 (1 – 4) | 3 (1.5 – 4) | 2 (1 – 3) | 3.5 (1 – 6) | 3 (1 – 4.5) | < 0.001 |
| **Defibrillation.** *n %* | 646 (60) | 14 (30) | 632 (62) | 4 (25) | 109 (43) | 10 (32) | 523 (68) | < 0.001 |
| **Admission lab –** *median; IQR*  **pH**  **lactate** *mmol/l*  **glucose** *mg/dl* | 7.19 (7.03 – 7.29) 7.4 (4.3 – 10.9) 228.5 (171– 314.75) | 7.13 (7.03–7.26)  10 (6.15 - 12.3)  215 (144-282.5) | 7.19 (7.03 – 7.29)  7.3 (4.3 – 10.85)  230 (172 – 315) | 7.10 (7.06 – 7.23)  9.9 (7.3-12.8)  228 (132 – 192) | 7.18 (7.00 – 7.33)  6.1 (3.3 – 10.1)  196 (148 – 286) | 7.15 (7.03 – 7.27)  10.05 (5.9 – 11.76)  208 (148 – 252.5) | 7.19 (7.05 – 7.28)  7.65 (4.6 – 11)  238 (181 – 323) | 0.81  < 0.05  0.07 |
| **Therapeutic hypothermia.** *n %* | 666 (62) | 18 (38) | 648 (63) | 3 (18) | 83 (33) | 15 (48) | 565 (74) | < 0.001 |
| **Re-Arrest <2h.** *n %* | 95 (9) | 3 (6) | 92 (9) | 0 (0) | 22 (9) | 3 (10) | 70 (9) | 0.54 |

*Abbreviations:* ROSC, Return of spontaneous circulation ; VT, Ventricular Tachycardia; VF, Ventricular Fibrillation ; PEA, Pulseless electrical activity; mg, milligram;
*Cirrhosis vs. no cirrhosis
